# Stunting Mediates the Association between Small-for-Gestational-Age and Postneonatal Mortality^1^,^2^,^3^

**DOI:** 10.3945/jn.116.235457

**Published:** 2016-10-12

**Authors:** Vanessa M Oddo, Parul Christian, Joanne Katz, Li Liu, Naoko Kozuki, Robert E Black, Robert Ntozini, Jean Humphrey

**Affiliations:** 4 Departments of International Health and; 5 Population, Family, and Reproductive Health, Johns Hopkins Bloomberg School of Public Health, Baltimore, MD;; 6 Bill & Melinda Gates Foundation, Seattle, WA; and; 7 ZVITAMBO Institute for Maternal and Child Health Research, Harare, Zimbabwe

**Keywords:** small for gestational age, postneonatal mortality, stunting, mediation, HIV

## Abstract

**Background:** In sub-Saharan Africa, one-third of all births are small for gestational age (SGA), and 4.4 million children are stunted; both conditions increase the risk of child mortality. SGA has also been shown to increase the risk of stunting.

**Objective:** We tested whether the association between SGA and postneonatal mortality is mediated by stunting.

**Methods:** We used longitudinal data from children aged 6 wk to 24 mo (*n* = 12,155) enrolled in the ZVITAMBO (Zimbabwe Vitamin A for Mothers and Babies) trial. HIV exposure was defined based on maternal HIV status at baseline. SGA was defined as birthweight <10th percentile of the INTERGROWTH-21st (International Fetal and Newborn Growth Consortium for the 21st Century) standards. We used a standard mediation approach by comparing the attenuation of the risk when the mediator was added to the model. We used Cox proportional hazards models first to regress SGA on postneonatal mortality, controlling for age. Stunting (length-for-age *z* score <−2) was then included in the model to test mediation.

**Results:** Approximately 20% of children were term SGA, and 23% were stunted before their last follow-up visit. In this cohort, 31% of children were exposed to HIV; the HIV-exposed group represented a pooled group of HIV-infected and HIV-exposed but uninfected children. Postneonatal mortality was significantly higher among children born SGA (HR: 1.5; 95% CI: 1.3, 1.7). This association was attenuated and not statistically significant when stunting was included in the model, suggesting a mediation effect (HR: 1.1; 95% CI: 0.91, 1.3). When stratified by HIV exposure status, we observed a significant attenuation of the risk, suggesting mediation, only among HIV-exposed children (model 1, HR: 1.3; 95% CI: 1.1, 1.6; model 2, HR: 1.1; 95% CI: 0.88, 1.3).

**Conclusions:** This analysis aids in investigating pathways that underlie an observed SGA-mortality relation and may inform survival interventions in undernourished settings.

## Introduction

Recent estimates of global child survival highlight the urgent need to accelerate progress in preventing child deaths (1). In sub-Saharan Africa, ∼26% of all births are small for gestational age (SGA)^8^ (birthweight <10th percentile of a sex- and gestational-age–specific population reference), and 4.4 million children are stunted [length-for-age *z* score (LAZ) and height-for-age *z* score (HAZ) <−2], putting children at increased risk of mortality (2–5).

**FIGURE 1:**
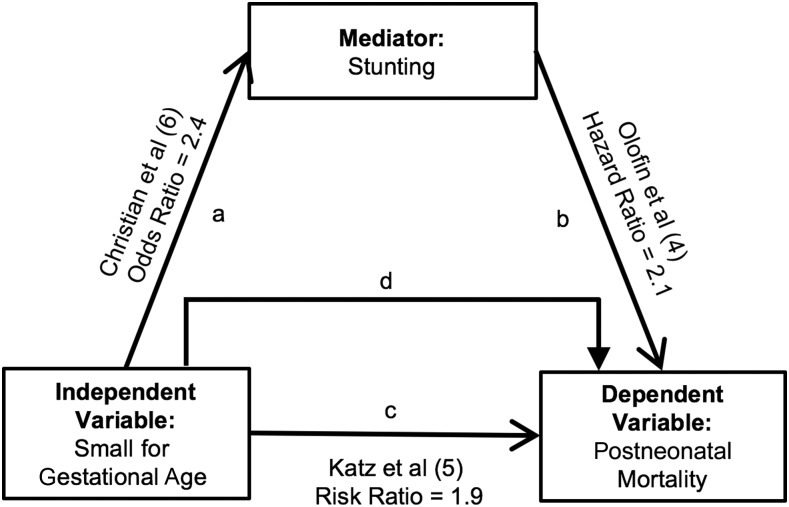
Hypothesized pathways of the association between small for gestational age and mortality among children aged 6 wk to 24 mo in the ZVITAMBO cohort. Small for gestational age was defined as the weight <10th percentile with the use of INTERGROWTH-21st standards (7, 8). INTERGROWTH-21st, International Fetal and Newborn Growth Consortium for the 21st Century; ZVITAMBO, Zimbabwe Vitamin A for Mothers and Babies.

Research has not yet linked SGA to postneonatal mortality via stunting. However, Christian et al. (6) provided strong evidence of a positive association between SGA and stunting in a recent meta-analysis of 19 longitudinal birth cohorts (**Figure 1**, pathway a). Term SGA was associated with a 2.4 times higher odds of stunting, whereas being both SGA and preterm (<37 wk gestation) was associated with a 4.5 times higher odds of stunting (6). Approximately one-fifth of childhood stunting was attributed to SGA in Christian et al. (6). Both conditions have a high prevalence in many low- and middle-income countries (9). Stunting in turn is also associated with higher mortality (Figure 1, pathway b). Results from Olofin et al. (4) indicate that the relation between stunting and all-cause mortality is present even for children aged 1 wk to 59 mo with an HAZ of −1 to −2. The risk significantly increases with stunting severity. Mortality hazards are 2 times higher among children who are stunted (HAZ <−2 to −3) and 5 times higher among children who are severely stunted (HAZ <−3) (4). Evidence also indicates that there is a strong positive association between SGA and all-cause infant mortality (Figure 1, pathway c). Katz et al. (5) reported that postneonatal mortality is ∼2 times higher among infants who are SGA than those who are appropriate for gestational age (AGA).

Mediation analyses aid in investigating potential pathways that underlie an observed relation between an exposure and outcome variable. Building on previous work that documents the SGA-stunting, stunting-mortality, and SGA-mortality relations, we compared the attenuation of the risk when the mediator was added to the model to test whether stunting mediates the association between SGA and postneonatal mortality (Figure 1, pathway d) (4–6). We hypothesized that the link between SGA and postneonatal mortality may be related in part to the increased risk of stunting among children born SGA. We used data from the ZVITAMBO (Zimbabwe Vitamin A for Mothers and Babies) trial, a birth cohort of postpartum mothers and their infants in Harare, Zimbabwe, in which birth and repeated postnatal anthropometry was conducted prospectively in the first 12–24 mo of life. ZVITAMBO characterized HIV status (unexposed, exposed) and growth in a population of increasing public health interest: HIV-exposed but uninfected (HEU) children. Understanding whether stunting mediates the association between SGA and postneonatal mortality is important for informing survival interventions in low- and middle-income countries.

## Methods

### 

#### Study population.

We used longitudinal data from ZVITAMBO. The ZVITAMBO protocol and primary outcomes have been described in detail elsewhere (9–12). Briefly, 14,110 postpartum mothers and their infants were enrolled within 96 h of delivery between November 1997 and January 2000 in Harare, Zimbabwe, to measure the independent and combined effects of neonatal and maternal vitamin A supplementation on mortality in infants born to HIV-negative mothers and on mother-to-child transmission of HIV and HIV-free survival among infants born to HIV-positive mothers (10). The trial was a 2 × 2 factorial design in which the interventions included mother treatment and infant treatment, mother treatment and infant placebo, mother placebo and infant treatment, and mother placebo and infant placebo, in which maternal and infant vitamin A treatment was 400,000 and 50,000 IU retinyl palmitate, respectively. Participants were eligible if neither mother nor infant had an acutely life-threatening condition, the infant was a singleton with a birthweight ≥1500 g, and the mother was available for follow-up. Questionnaires, hospital records, and direct measurements were used to collect data at baseline. Mother-child dyads were followed at 6 wk, 3 mo, and every 3 mo until 12–24 mo at a study clinic or at home. The trial originally planned to follow all HIV-positive mothers and their children and a random sample of an approximately equal number of HIV-negative mothers and their children to 24 mo postpartum and to follow the remaining HIV-negative mother-child dyads to 12 mo postpartum. However, in June 2000, economic issues made it necessary to discontinue the second year of follow-up, so dyads were administratively censored at their 12-mo visit or at their next 3-mo visit if already beyond their 12-mo visit. At baseline and all follow-up visits, weight (to the nearest 0.01 kg) and length (to the nearest 0.1 cm) were measured with the use of a Seca 727 electronic scale and ShorrBoard length board during each visit (13). Vital status (defined as dead or alive) was recorded at each follow-up visit. Mothers provided written informed consent for participation in the study as well as their children’s participation.

In our analyses, we used the most proximate anthropometric measurement before the last observed measurement of vital status to establish the temporality of the SGA stunting-mortality relation. The first follow-up visit was at 6 wk; therefore, we excluded children who died before 6 wk of age (*n* = 168) and children whose only follow-up visit was at 6 wk (*n* = 266). Those missing gestational age at delivery (*n* = 150), sex (*n* = 6), birthweight (*n* = 46), length, weight, and/or age (*n* = 1305) or had extreme values for LAZ (LAZ <−6 or LAZ >6) (*n* = 14) were also excluded. The analyses included 12,155 children. Approximately 39%, 10%, 8%, 8%, and 31% of children were administratively censored at 12, 15, 18, 21, and 24 mo, respectively. The Johns Hopkins Bloomberg School of Public Health Institutional Review Board deemed this analysis of deidentified secondary data as exempt.

#### Primary variables of interest.

SGA was defined as birthweight <10th percentile with the use of the sex- and gestational-age–specific International Fetal and Newborn Growth Consortium for the 21st Century (INTERGROWTH-21st) birthweight standards (7, 8). Newborns whose gestational age at delivery was <37 wk were categorized as preterm. Age (mo) was defined as the number of days between date of birth and date of anthropometric measurement. LAZ was estimated according to the 2006 WHO Multicentre Growth Standards, and stunting was defined as LAZ <−2 (14).

#### Mediation analysis.

Recent work by Valeri and VanderWeele (15) highlighted the conditions under which traditional mediation approaches (i.e., difference, product) are valid for proportional hazards models; in cases in which the exposure-mediator interaction is not significant and the outcome is binary and rare, a single-mediator approach is appropriate (16). Building upon prior literature, we followed this stepwise strategy with the use of Cox proportional hazards regression models. Model 1 assessed the overall effect between SGA and postneonatal mortality, and model 2 assessed the attenuation of the SGA-mortality effect after controlling for stunting at the most proximate anthropometric measure before the last vital status observed to establish temporality. All models were adjusted for age at the most proximate anthropometric measurement. Mediation was inferred when the association between SGA and postneonatal mortality was attenuated (comparing model 1 to model 2) and no longer statistically significant in model 2.

We further explored the hypothesized mediation effect in 2 stratified analyses. First, the literature has suggested that the mediation effect of stunting on the association between SGA and mortality may vary by the age of the child at death (17, 18). Accordingly, analyses were stratified by 3 age groups: 6 wk to 6 mo, 6–12 mo, and 12–24 mo. Second, in this HIV-endemic cohort, mortality was largely determined by HIV status (19). Therefore, we also stratified by children’s exposure to HIV. This specification uses the mother’s HIV status assessed at baseline. Although prenatal and postnatal growth is known to differ between HEU and HIV-infected children, analyses suggested that the mediation effect is homogenous between these two groups. Therefore, the HIV-exposed group for this analysis represents a pooled group of HEU and HIV-infected children.

In robustness checks, we examined whether our results changed when *1*) stratifying by sex, *2*) controlling for wasting (weight-for-height *z* score <−2) at the most proximate anthropometric measure before the last vital status, and *3*) removing age as a confounder. In this cohort, the risk of mortality varied by the timing of infection (19). Therefore, additional analyses included stratifying by the timing of infection (HEU or HIV-infected in utero, intrapartum, or postnatally). We also assessed the robustness of our results when defining exposure to HIV based on mothers’ last available test result during the first 12 mo of follow-up. To further test whether stunting is on the causal pathway and not an effect modifier of the SGA-mortality association, we explored the association between SGA term (compared with AGA term) and mortality stratified by stunting status at 3 mo of age. We set α to 0.05 and performed statistical analyses in Stata version 14.1 (StataCorp LP).

## Results

Children (*n* = 12,155) were followed for a mean of 70 wk. To estimate the attenuation of risk when stunting was included in the model, we used the most proximate anthropometric measure before the last vital status observed. For ∼85% of our sample, the most proximate measure was within 3 mo of the last observed vital status.

Child, maternal, and household baseline characteristics are presented in **Table 1**. In total, ∼23% of the study children were stunted before their last observed vital status, and 31% (*n* = 3800) were HIV-exposed at baseline. The infant mortality rate was 59/1000 births. The prevalence of SGA term was 20%. An additional 1% of the sample was SGA and preterm (<37 wk).

**TABLE 1:** Sample characteristics of children aged 6 wk to 24 mo in the ZVITAMBO cohort^1^

	*n*	Values
Age,^2^ mo	12,155	11.1 ± 0.05
Sex, %		
Males	6237	51
Females	5918	49
Proximal stunting,^3^ %	2842	23
SGA term status,^4^ %		
Term AGA	8913	73
Term SGA	2371	20
Preterm AGA	698	5.7
Preterm SGA	173	1.4
HIV exposure status,^5^ %		
Exposed	3800	31
Unexposed	8331	69
Postneonatal mortality (6 wk to 24 mo),^6^ *n*/1000	717	59
Postneonatal mortality by child HIV exposure status,^5^^,^^6^ *n*/1000		
Exposed	566	149
Unexposed	149	18

1 Values represent the mean ± SD, percentage, or rate per 1000 live births as appropriate (missing vital status, *n* = 8; missing or no result for HIV exposure, *n* = 24). AGA, appropriate for gestational age; INTERGROWTH-21st, International Fetal and Newborn Growth Consortium for the 21st Century; SGA, small for gestational age; ZVITAMBO, Zimbabwe Vitamin A for Mothers and Babies.

2 Age at the most proximate anthropometric measurement before the last observed measurement of vital status.

3 Stunting was defined as a height-for-age *z* score <−2 according to the 2006 WHO Multicentre Growth Standards and reflects stunting at the most proximate anthropometric measurement before the last observed measurement of vital status (14).

4 SGA was defined as weight <10th percentile with the use of INTERGROWTH-21st standards. Infants whose gestational age was <37 wk were categorized as preterm (7, 8).

5 Exposure to HIV was defined based on maternal HIV status at baseline. HIV-exposed represents a pooled group of HIV-exposed but uninfected children and HIV-infected children.

6 Represents the mortality rate among the analytic sample.

The relation between SGA and postneonatal mortality was mediated through stunting in our primary model specification (**Table 2**). Model 1 showed that SGA was associated with a significantly higher hazard of postneonatal mortality (HR: 1.5; 95% CI: 1.3, 1.7). The association between SGA and postneonatal mortality was attenuated and no longer statistically significant when stunting was included in model 2 (HR: 1.1; 95% CI: 0.91, 1.3). In stratified analyses, model 1 indicated that SGA was associated with a 1.8 times (95% CI: 1.3, 2.6) higher hazard of postneonatal mortality among children aged 6 wk to 6 mo. In model 2, the association was attenuated to 1.3 (95% CI: 0.88, 1.9) when proximate stunting was included and was no longer statistically significant. Similarly, among children aged 6–12 mo (HR: 1.6; 95% CI: 1.1, 2.1) and 12–24 mo (HR: 1.8; 95% CI: 1.2, 2.7), model 1 showed SGA was associated with a higher risk of postneonatal mortality. For both age groups, the association was attenuated and no longer significant when stunting was included in model 2 (6–12 mo, HR: 1.1; 95% CI: 0.82, 1.6 and 12–24 mo, HR: 1.3; 95% CI: 0.82, 2.0), suggesting the SGA-mortality relation was mediated by stunting.

**TABLE 2:** The SGA-mortality association mediated by stunting among children aged 6 wk to 24 mo in the ZVITAMBO cohort^1^

	Overall (*n* = 12,155)	≥6 wk to <6 mo (*n* = 8090)	≥6 to <12 mo (*n* = 7291)	≥12 to ≤24 mo (*n* = 3626)
Model 1				
SGA^2^	1.5 (1.3, 1.7)*	1.8 (1.3, 2.6)*	1.6 (1.1, 2.1)*	1.8 (1.2, 2.7)*
Model 2				
SGA^2^	1.1 (0.91, 1.3)	1.3 (0.88, 1.9)	1.1 (0.82, 1.6)	1.3 (0.82, 2.0)
Stunting^3^	2.7 (2.3, 3.2)*	2.6 (1.8, 3.7)*	3.9 (2.9, 5.2)*	5.4 (3.6, 8.8)*

1 Values are HRs (95% CIs) estimated with the use of Cox regression. All models were controlled for age during the anthropometric measurement. *P < 0.05. INTERGROWTH-21st, International Fetal and Newborn Growth Consortium for the 21st Century; SGA, small for gestational age; ZVITAMBO, Zimbabwe Vitamin A for Mothers and Babies.

2 SGA was defined as weight <10th percentile with the use of INTERGROWTH-21st standards (7, 8).

3 Stunting was defined as height-for-age *z* score <−2 according to the 2006 WHO Multicentre Growth Standards and reflects stunting at the most proximate anthropometric measurement before the last observed measurement of vital status (14).

When stratified by HIV exposure status, we observed a significant attenuation of risk, suggesting mediation, only among HIV-exposed children. However, the point estimates among those not exposed to HIV followed a similar trend (**Table 3**). Among HIV-exposed children, the significant SGA postneonatal-mortality relation in model 1 (HR: 1.3; 95% CI: 1.1, 1.6) was attenuated and no longer significant (HR: 1.1; 95% CI: 0.88, 1.3) when stunting was added as a covariate in model 2. Among HIV-unexposed children, the association between mortality and SGA was not significant in model 1 (HR: 1.1; 95% CI: 0.77, 1.7) or model 2 (HR: 0.87; 95% CI: 0.58, 1.3).

**TABLE 3:** The SGA-mortality association mediated by stunting by HIV exposure status among children aged 6 wk to 24 mo in the ZVITAMBO cohort^1^

	HIV-exposed children^2^^,^^3^ (*n* = 3800)	HIV-unexposed children^2^ (*n* = 8331)
Model 1		
SGA^4^	1.3 (1.1, 1.6)*	1.1 (0.77, 1.7)
Model 2		
SGA^4^	1.1 (0.88, 1.3)	0.87 (0.58, 1.3)
Stunting^5^	2.1 (1.7, 2.4)*	2.8 (2.0, 4.0)*

1 Values are HRs (95% CIs) estimated with the use of Cox regression. All models were controlled for age during the anthropometric measurement. *P < 0.05. INTERGROWTH-21st, International Fetal and Newborn Growth Consortium for the 21st Century; SGA, small for gestational age; ZVITAMBO, Zimbabwe Vitamin A for Mothers and Babies.

2 Exposure to HIV was defined based on maternal HIV status at baseline. Estimates excluded children of women whose test gave an indeterminate result or whose test was missing at baseline (*n* = 24).

3 HIV-exposed represents a pooled group of HIV-exposed but uninfected children and HIV-infected children.

4 SGA was defined as weight <10th percentile with the use of INTERGROWTH-21st standards (7, 8).

5 Stunting was defined as height-for-age *z* score <−2 according to the 2006 WHO Multicentre Growth Standards and reflects stunting at the most proximate anthropometric measurement before the last observed measurement of vital status (14).

Our results largely remained consistent in sensitivity analyses. We observed a significant attenuation of risk, suggesting mediation, both in males and females (**Supplemental Table 1**), when including wasting as a confounder (**Supplemental Table 2**), and when age was removed as a confounder (**Supplemental Table 3**). We did not observe a mediation effect among subgroups (HEU compared with HIV-infected) when results were stratified by the timing of HIV infection among children (**Supplemental Table 4**); however, we may have been underpowered to detect the association between SGA and mortality by the timing of infection. Results were unchanged in magnitude, direction, and significance when defining HIV exposure based on maternal HIV status at the last known test result (**Supplemental Table 5**). We did not observe a significant association between SGA term (compared with AGA term) and mortality when results were stratified by stunting status at 3 mo of age (**Supplemental Table 6**).

## Discussion

In these analyses, we interpreted mediation by comparing the attenuation of the risk when the mediator (i.e., stunting) was added to the model (16). Our results indicate that being born SGA was associated with a 1.5 times higher hazard of postneonatal mortality, but the hazard was attenuated to 1.1 and was no longer statistically significant when stunting was included in the model, suggesting a mediation effect. We did not see differences in the SGA-mortality relation when results were stratified by stunting status (Supplemental Table 6), which is consistent with our hypothesis that stunting is on the casual pathway and not an effect measure modifier of the SGA-mortality association. These findings suggest that a high prevalence of SGA in HIV-endemic undernourished populations leads to greater mortality through a pathway somewhat mediated by stunting. This is consistent with evidence from other HIV-endemic cohorts that have suggested that many HIV-exposed children are born SGA (and thus that growth failure begins in utero) and work that has previously shown that children born SGA have a 2- to 3-fold higher risk of stunting in childhood (11, 20–24). We also highlight that SGA was associated with long-term mortality in children mediated through stunting. This finding is new to our knowledge relative to previous literature on the SGA-mortality association and contributes to the growing body of evidence that highlights the importance of nutrition in utero and a life-course perspective to intervention implementation (6, 25–27).

Our results are consistent with previous literature that has suggested that the magnitude of the association between stunting and postneonatal mortality increases with increasing age because of the increasing risk of linear growth faltering by age, with peak stunting rates occurring between the ages of 24 and 36 mo (25, 28). Despite similar growth trajectories, children born SGA may be more stunted later as a result of not catching up with children born AGA. We speculate that stunting is a mediator among children aged 6–24 mo given this cumulative effect of SGA and stunting.

Undernutrition is synergistic with infectious diseases in increasing the risk of childhood mortality (26). Stunted children are more susceptible to infections and vice versa, likely leading to an increased risk of postneonatal mortality. In this HIV-endemic sample, we interpret that stunting was a mediator of the SGA postneonatal-mortality relation among those children who were exposed to HIV (i.e., mothers were HIV-positive) but not for those who were unexposed. This is consistent with other findings from this cohort that have suggested that more linear growth failure occurs when children are exposed to HIV than those who are unexposed (AH Omoni, J Humphrey, C Evans, A Prendergast, L Moulton, and P Christian, unpublished data, 2016). Among the HIV-exposed children in ZVITAMBO, inflammatory markers (e.g., C-reactive protein and α-acid glycoprotein) were associated with stunting (26). Similarly, Evans et al. (29) found that immune activation and inflammation are likely key drivers of postnatal growth failure among HEU compared with unexposed children. It is possible that stunting may play a stronger mediating role between SGA and postneonatal mortality among HIV-exposed immunocompromised children than among HIV-unexposed children. It is also plausible that with increasing age, the more proximal risk factor (stunting) becomes a stronger predictor of postneonatal mortality than the more distal risk factor (SGA).

Our data analyses and approach have several strengths. We included a large birth cohort followed longitudinally, and regular follow-up visits allowed us to establish the temporality of the SGA-stunting postneonatal-mortality relation. Limitations of this study also warrant consideration. This analysis may have been underpowered to detect a mediation effect of the SGA postneonatal-mortality association because neonatal deaths were excluded, which is when ∼30% of deaths occurred in the HIV-unexposed strata of children (but only ∼10% of deaths among HIV-exposed). However, the risk of mortality was also attenuated when stunting was added to the model among HIV-unexposed children. There was sample selection bias resulting from attrition within the cohort, although most of this bias was caused by systemic administrative censoring. Mortality may result in a potential survival bias, which would mean that our estimates underestimated the mediation effect of stunting on the association between SGA and mortality. Finally, the exclusion of mothers and infants with life-threatening conditions and infants <1500 g from the ZVITAMBO trial potentially reduced the numbers of SGA and preterm infants in the sample and may have introduced selection bias. In addition, among children, HIV status was measured after follow-up; samples were collected from each child at regular intervals, and if the last available sample was positive for HIV, samples that had been collected at younger ages were retrospectively tested to determine the timing of the infection. The timing of seroconversion is only available within a 3-mo interval, and it is plausible that children could be misclassified as HEU instead of postnatally infected. However, sensitivity analyses, which excluded potentially misclassified children, indicated that the results remained unchanged (10). Finally, our results may be less generalizable to non-HIV–affected contexts.

Building upon prior work that has documented the SGA-mortality, SGA-stunting, and stunting-mortality relations and the growing body of evidence that suggests that child growth and mortality originate during fetal life in undernourished contexts, we demonstrated that the association between SGA and child mortality is mediated by stunting. These analyses inform the well-documented relation between SGA and postneonatal mortality, which identifies a vulnerable group of children who are born with fetal growth restriction and have an increased risk of future mortality (beyond immediate risk) mediated via stunting. These analyses also further our understanding of the SGA-mortality relation among HIV-exposed children, including HEU children—a population of interest as the coverage of effective interventions to prevent the mother-to-child transmission of HIV increases. A better understanding of this relation is important for informing future programmatic work, including providing appropriate interventions. Future work should consider exploring stunting as a mediator of the relation between SGA and mortality in populations without HIV and in other undernourished settings.

## Footnotes

8 Abbreviations used: AGA, appropriate for gestational age; HAZ, height-for-age *z* score; HEU, HIV-exposed but uninfected children; LAZ, length-for-age *z* score; SGA, small for gestational age; ZVITAMBO, Zimbabwe Vitamin A for Mothers and Babies.
